# Retinoic acid induces differentiation of cochlear neural progenitor cells into hair cells

**DOI:** 10.1016/j.bjorl.2021.01.005

**Published:** 2021-02-21

**Authors:** Minyun Chen, Jianmin Huang

**Affiliations:** a The Second Affiliated Hospital of Fujian Medical University, Department of Otolaryngology, Fujian, China; b Fujian Medical University Union Hospital, Department of Otolaryngology, Fujian, China

**Keywords:** Cochlear neural progenitor cells, Math1, MyosinVIIa, Retinoic acid

## Abstract

**Introduction:**

Inner ear progenitor cells have the potential for multi-directional differentiation. Retinoic acid is an important requirement for the development of the inner ear. Blocking the Curtyr's retinoic acid signaling pathway can significantly reduce the number of hair cells. Therefore, we believe that retinoic acid may induce the regeneration of inner ear hair cells.

**Objective:**

To investigate whether the cochlear neural progenitor cells maintain the characteristics of stem cells during recovery and subculture, whether retinoic acid can induce cochlear neural progenitor cells into hair cells in vitro, and whether retinoic acid promotes or inhibits the proliferation of cochlear neural progenitor cells during differentiation.

**Methods:**

Cochlear neural progenitor cells were cultured and induced in DMEM/F12 + RA (10^−6^ M) and then detected the expressions of hair cell markers (Math1 and MyosinVIIa) by immunofluorescence cytochemistry and realtime-polymerase chain reaction, and the proliferation of cochlear neural progenitor cells was detected by Brdu.

**Results:**

The nestin of cochlear neural progenitor cells was positively expressed. The ratios of Math1-positive cells in the control group and experimental group were 1.5% and 63%, respectively; the ratios of MyosinVIIa-positive cells in the control group and experimental group were 0.96% and 56%, respectively (*p* < 0.05). The ratios of Brdu^+^-labeled cells in retinoic acid group, group PBS, and group FBS were 20.6%, 29.9%, and 54.3%, respectively; however, the proliferation rate in the experimental group decreased.

**Conclusion:**

Retinoic acid can promote cochlear neural progenitor cells to differentiate into the hair cells.

## Introduction

The number of patients with sensorineural deafness is increasing, and electronic cochlear implantation as a substitute for artificial inner ear has achieved certain effects on restoring the hearing; however, the resolution of cochlear implant for sound can’t reach the normal cochlear level.^1^ In addition, due to the trauma and risks of cochlear implantation, its long-term effects still need further evaluation. Therefore, exploring effective methods for the regeneration and functional recovery of inner ear hair cells is still an important research direction for sensorineural hearing loss studies. Unlike non-mammalian,^2^ mammalian hair cells have very limited regenerative capabilities that are far from functional recovery.^3^, ^4^ In recent years, stem cell transplantation has become a research hotspot for sensorineural hearing loss. Embryonic stem cells,^5^, ^6^ bone marrow mesenchymal stem cells,^7^ or CNPs (cochlear neural progenitor cells)^8^ have all been applied to the study of hair cell regeneration and also expressed certain markers of hair cells. Stem cells can be divided into primary cells and cell lines. Primary cells have biological characteristics closer to living cells, but their cell viability is weak, and some of them can’t survive for three generations; cell lines have stable biological properties, are more convenient for studies, and can be much easily used for exploring the effects of single factor on cell differentiation. In this study, retinoic acid (RA) was used to induce the differentiation of inner ear progenitor cells. The inner ear progenitor cells used in this study were the cell lines derived from mouse auditory sensory epithelial cells, which were established in 2003 by Ozeki et al.^9^ CNPs can re-enter the cell cycle under the action of specific cell differentiation factors and signaling pathways and may differentiate into hair cells. LIN JL used Sonic Hedgehog (SHH), epidermal growth factor (EGF), RA, and brain-derived neurotrophic factor (BDNF) to co-induce CNPs, and found that it can induce the inner ear progenitor cells to differentiate into hair cells.^10^ Malgrange used SHH to directly induce CNPs to differentiate into hair cells,^11^ indicating that CNPs are the precursor cells of hair cells and are an ideal source of stem cells.

Inner ear progenitor cells can differentiate into hair cells under a variety of inducing factors, but the single-factor role is unclear. The reason why RA was used to induce the cell differentiation in this study depended on its characteristics. RA is a low-molecular-weight lipophilic molecule that can be easily obtained and is one of the important conditions for the inner ear development. RA can regulate protein expression at the gene level.^3^ It can regulate the expression of the cytoskeletal protein MyosinVIIa at the gene level, which is one of the markers of hair cells.^12^ During the regeneration of fish and chicken^13^ hair cells, RA promotes supporting cells to differentiate into hair cells. Differentiation and regeneration into hair cells are mainly through two ways: (1) The sensory precursor cells re-enter the cell cycle after being induced and activated, followed by proliferation through mitosis, and further differentiation into hair cells and support cells; (2) The sensory precursor cells directly differentiate into the hair cells.^14^ RA has certain protective effects on hearing.^15^, ^16^ Blocking the RA signal pathway of the organ of Corti reveals that the number of hair cells is significantly reduced.^17^ Therefore, we consider that it also has certain effects on the differentiation of hair cells.

In this study, RA was used for induction to explore its roles in the differentiation of inner ear progenitor cells into hair cells.

## Methods

### Culture and induction of CNPs

The inner ear progenitor cells used in this study were provided by the laboratory of University of Minnesota, which is a cell line of mouse auditory sensory epithelial cells, was firstly established in 2003 by Ozeki et al.,^9^ has multidirectional differentiation potential,^18^ and has relatively high homology with cochlear auditory cells.

The inner ear progenitor cells were firstly removed from a cryopreservation tube (University of Minnesota, Minnesota, USA) and cultured in the medium consisting of DMEM/F12 (Hyclone, Chicago, USA) +1% N2 (peprotech, New Jersey, USA)) +EGF (10 ng/mL) (peprotech, New Jersey, USA) +Bfgf (10 ng/mL) (peprotech, New Jersey, USA) with the culture medium being changed every other day. One Nikon inverted microscope (Nikon, Shanghai, China) was used to observe the cell morphology and growth status, and the expression of nestin in the cells were detected using the cellular immunochemical method. The cells in good growth status were then sampled, rinsed with PBS, and divided into the experimental group (EXP) and the control group (CON) for further cultivation in 12-well plates. The cells in group EXP were induced with DMEM/F12 + RA (10–6 M) (Sigma, USA), and the cells in group CON were cultured with DMEM/F12 + DMSO (0.11 mg/mL) (Sigma, USA); the medium was changed every day. After 3 days, the expressions of hair cell marker proteins Math1 and MyosinVIIa were detected by immunofluorescence cytochemistry.

### Immunofluorescence cytochemistry

After having aspirated the cell culture medium, the residue was rinsed with PBS 3-times * 2 min, followed by drying the residue liquid, fixation in 100 μL of 4% paraformaldehyde for 20 min, PBS rinsing 3-times * 2 min, drying, 15-min cell membrane perforation using 100 μL of 0.3% triton solution, PBS rinsing 3-times * 2 min, and drying; 100 μL of 10% goat serum was then added for 30 min blockage at 37 °C; the goat serum was then shaken away (no rinsing). 100 μL of rabbit anti-mouse nestin (Bioss, Beijing, China) (dilution 1:100) was then added for overnight culture at 4 °C in a humidifier (group CON was added PBS for contrast). After the mixture was rewarmed on the next day for 45 min, it was rinsed with PBS 3-times * 2 min, followed by drying, 15 min culture with 100 μL polymer adjuvant at 37 °C, PBS rinsing 3 times * 2 min, drying, 15 min culture with 100 uL of HRP-labeled goat anti-rabbit IgG (Nakasugi Jinqiao, Beijing, China) at 37 °C, PBS rinsing 3 times * 2 min, drying, 5 min culture with 100 μL of DAB solution in darkness at room temperature, tap water rinsing, drying, 2 min staining with 100 μL of hematoxylin solution, distilled water rinsing, drying, and observation under an inverted microscope.

### Real time Polymerase Chain Reaction (RT-PCR)

Trizol was used to extract the total RNA, detecting the concentration and purity using a microplate reader. The purity of the total RNA was OD > 1.8, and the integrity of RNA was detected by RNA agarose gel electrophoresis. The total RNA was then reversely transcribed into cDNA using a reverse transcription kit, followed by PCR amplification. The primers were synthesized by Shanghai Shengong.

Primers:

Math1(269 bp): upstream: 5^,^-CCAGGGTGAGCTGGTAAGG-3; downstream: 5,-CGTTGTTGAAGGACGGGAT-3; Myosin7a(628 bp): upstream: 5,-AAGCACCTGCTCCTGCTCGTCCACG-3; downstream: 5,-CTCCCTCTACATCGCTCTGTTCG-3, GAPDH (462 bp): upstream: 5,-TGCTGTCCCTGTATGCCTCT-3, downstream: 5^,^-GGTCTTTACGGATGTCAACG-3^,^

PCR reaction conditions were as follows: Math1: pre-denaturation at 94 °C for 5-min, denaturation at 94 °C for 30 s, annealing at 59.8 °C for 30 s, 72 °C for 30 s, and primer extension at 72 °C for 5 min, for a total of 40 cycles. The reaction conditions of Myosin7a and GAPDH were the same as those of Math1, but the annealing temperatures were 64 °C and 57 °C, respectively.

The electrophoresis results were observed with a gel imaging developer.

### Intracellular expression of Math1 and MyosinVIIa after induction

The cells in good growth status were harvested, washed with PBS, divided into group EXP and group CON, and then cultured in 12 well plates. The cells in group EXP were induced with DMEM/F12 + RA (10–6 M), and those in group CON were treated with DMEM/F12 + DMSO (0.11 mg/mL): the medium was changed every day. After 3 days, the expressions of hair cell marker proteins (Math1 and MyosinVIIa) were detected by immunofluorescence cytochemistry. The procedures were as follows: after sucking away the cell culture medium, the cells were rinsed with PBS 3 times * 2 min, followed by 20 min fixation with 100 μL of 4% paraformaldehyde, PBS rinsing 3 times * 2-min, 15 min cell membrane perforation using 100 μL of 0.3% triton solution, PBS rinsing 3 times * 2 min; 100 μL of 10% goat serum was then added for 30 min blockage at 37 °C; the goat serum was then shaken away (no rinsing); 80 μL of rabbit anti-mouse Math1 (Sigma, USA) (dilution 1:100) and 80 μL of rabbit anti-rat Myosin7a were then added for overnight culture at 4 °C in a humidifier (group CON was added PBS for contrast) After the mixture was rewarmed on the next day for 45 min, it was rinsed with PBS 3 times * 2 min. with all the operations performed in darkness. Later, the cells were added dropwise to 100 μL of Tric-labeled goat anti-rabbit IgG (ZSGB-BIO, Beijing, China) for 30 min incubation at 37 °C, followed by PBS rinsing 3 times * 2 min, 2 min incubation with DAPI solution at room temperature (in darkness), PBS rinsing 3 times * 2 min, and observation under a fluorescent inverted microscope. Five independent visual fields were selected for cell counting in each group, and the positive cell rate was calculated for statistical analysis.

### Brdu test

The cell suspension (at a concentration of 106 cells/mL) was inoculated into a 6well plate for one day. On the next day, the wall- adherent cells were harvested and divided into three groups: the experiment group (RA), the negative control group (Ctrl-), and the positive control group (Ctrl + ). Group RA was cultured with DMEM/F12 + RA (10–6 M) + Brdu (10 μg/mL), Group Ctrl- was cultured in DMEM/F12 + DMSO (0.11 mL/mL) + Brdu (10 μg/mL), and group Ctrl+ was cultured in DMEM/F12 + 10% FBS + Brdu (10 μg/mL); the culture lasted 48 h, and the culture medium was changed every day. Cellular immunochemical test was performed after 2 days. The method of immunohistochemistry was the same as that of nestin: denatured the nucleic acid with HCl before adding peroxide. The primary antibody was anti-mouse Brdu (Sigma, USA) (dilution 1:40), and the secondary antibody was HRP-labeled mouse anti-mouse IgG (ZSGB-BIO, Beijing, China). The results were observed under an inverted microscope. Six independent visual fields were selected in each group for cell counting, and the Brdu+ cell rate was calculated for statistical analysis.

### Statistical analysis

The results were expressed as $\bar{x}\pm \text{s}$, and the statistical analysis was performed with SPSS17.0. The differences between groups were analyzed by One-Way ANOVA. The LED (L) method was used when the variance was homogeneous, and the Dunnett's T3 method was used when the variance was non-homogeneous. The difference was considered to be statistically significant at *p* < 0.05. GraphPad Prism 5 software was used for mapping.

## Results

### Growth process of CNPs

**Figure 1:**
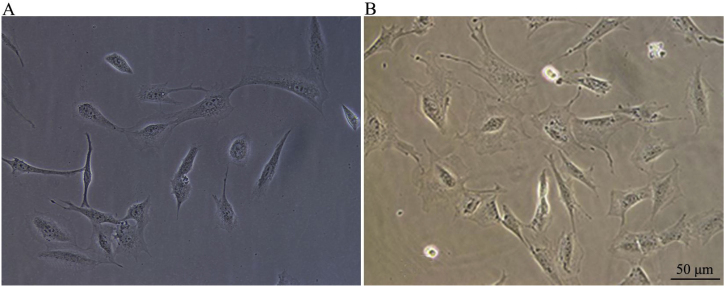
A, 24 h after passage of CNPs. B, 48 h after passage of CNPs (×200). The cell morphology of CNPs after 24 h and 48 h culture under an inverted microscope.

After thawing, the CNPs shrunk into round small cell spheres and became culture plate wall-adherent about half an hour after inoculation. Cells died and floated on the medium surface after 24 h. The survived cells all exhibited wall adherent. The cell shape was relatively simple and mostly spindle-shaped. Some cells exhibited a few cell protrusions (Fig. 1A). After 48 h, the cell morphologies were diversified, exhibiting oval, fusiform (main), or polygonal shapes. Cell protrusions grew further (Fig. 1B).

### Expression of specific marker of neural stem cells (nestin)

**Figure 2:**
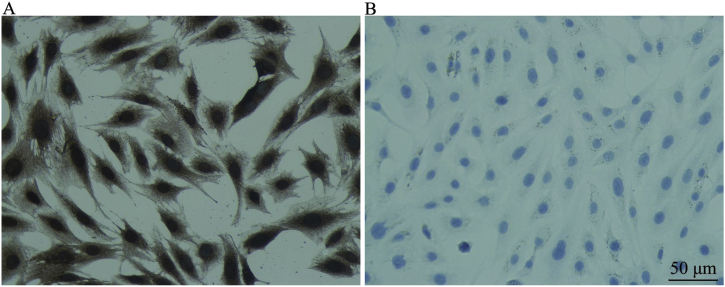
Detection of nestin in CNPs (cell immunochemical staining) (×200). A, Group EXP for detection of nestin in CNPs, and B, Group CON. A, Identification of nestin expression in CNPs by cellular immunochemical staining. The staining is in the cytoplasm. The positive staining is brownish yellow, and the negative is not stained. B, Nucleus staining of CNPs, and the nuclei are stained blue with hematoxylin.

In CNPs, the marker of neural stem cells was positively expressed. The inner ear progenitor cells were undifferentiated, which belong to the neural stem cells and are conducive to inducing differentiation (Fig. 2).

### RT-PCR

**Figure 3:**
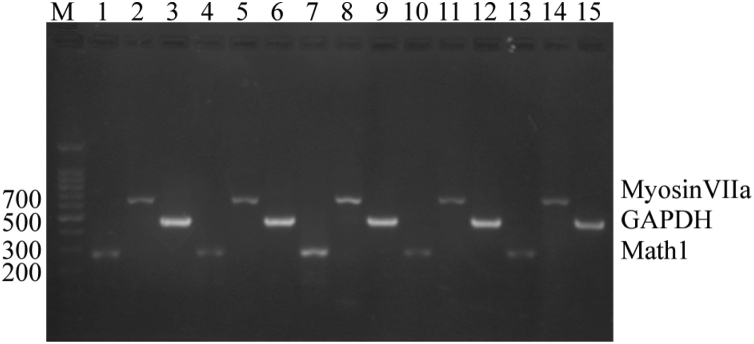
CNPs after induced differentiation by RT-PCR. Lane 1–16 refers to Marker; ctrl -Math1, MyosinVIIa, GAPDH, day 2 ctrl -Math1, MyosinVIIa, GAPDH, day 2 Math1, MyosinVIIa, GAPDH, day 3 ctrl -Math1, MyosinVIIa, GAPDH, day 3 Math1, MyosinVIIa, and GAPDH, respectively.

From the figure, it can be seen that the expressions of Math1 and Myosin7a gene can be seen in both group CON and group EXP. After 3 days of induction, the expressions of these two genes in group EXP were significantly enhanced than group CON (Fig. 3).

### Immunofluorescence staining

**Figure 4:**
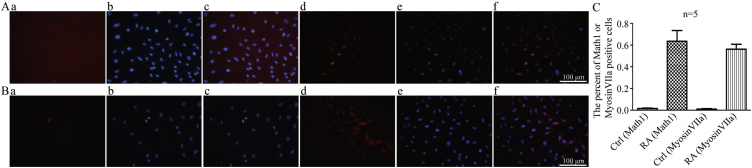
A, Immunofluorescence staining of Math1 in CNPs after differentiation induction (×200). (a and d) are Math1 staining (the stained nuclei exhibit red fluorescence); (b and e) are DAPI-stained nuclei (the stained nuclei exhibit blue fluorescence); (c and f) combine the first two images. B, Immunofluorescence staining of MyosinVIIa in CNPs after differentiation induction. (a and d) are MyosinVIIa staining (the stained cytoplasm exhibits red fluorescence); (b and e) are DAPI-stained nuclei (the stained nuclei exhibit blue fluorescence); (c and f) combine the first two images. C, Proportion of positively stained hair cell markers in CNPs after differentiation induction.

The rates of Math1-positive cells in group CON and group EXP were 1.5% and 63% (Fig. 4A and C), respectively, and the rates of MyosinVIIa-positive cells in group CON and group EXP were 0.96% and 56% (Fig. 4 B and C), respectively, *p* < 0.05, indicating that exogenous RA promotes the formation of Math1 and MyosinVIIa proteins in CNPs, so CNPs can gradually differentiate into hair cells.

### Proliferation detection

**Figure 5:**
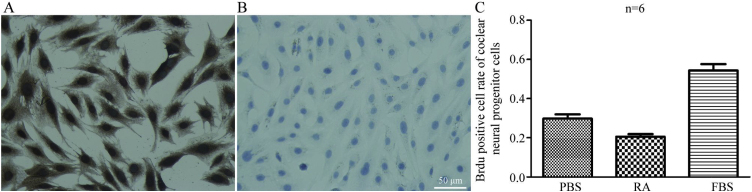
A, Brdu test of CNPs (cell immunochemical staining) (×400). Brdu test of CNPs, (a) Group ctrl-; (b) Group RA; (c) Group ctrl+. The nuclei of positive cells exhibited brown particles, and the nuclei were stained blue with hematoxylin. B, Ratio of Brdu+ cells in CNPs.

The nuclei of the positive cells exhibited brown particles, and the nuclei were stained blue with hematoxylin (Fig. 5A).

The staining rates of Brdu+ cells in group RA, group ctrl-, and group ctrl+ were 20.6%, 29.9%, and 54.3%, respectively, *p* < 0.05, indicating statistical significance among the three groups (Fig. 5B).

## Discussion

In this study, RA was used to induce CNPs to differentiate into hair cells. Cells with the markers of hair cells (myosinVIIa and Math1), as well as hair bundles, can be defined as hair cells.^19^ Myosin is a molecular movement protein, and MyosinVIIA is mainly located in cilia and microvilli; as a late product of hair cells, it is one of the markers for the identification of hair cells.^20^, ^21^

Math1 is a homologue of the Drosophila atomal gene, which can effectively promote the growth and development of hair cells, as well as induce the regeneration of hair cells.^22^, ^23^ It can appear at the early stage of hair cell generation, and is also a special gene that can promote the differentiation and maturity of hair cells.^10^ In this study, DMEM/F12 + RA was used as an inducer to induce CNPs. After 3 day induction, the markers of hair cells were detected from the gene and protein levels. The results show that the expressions of myosinVIIa and Math1 genes increased after 3 days of induction, and the expressions of myosinVIIa and Math1 proteins were also detected by cellular immunofluorescence, which further confirmed that RA can promote the differentiation of CNPs into hair cells. Other studies also have found that CNPs have the potential to replace degenerated nerve connections.^24^

The best feature of the culture process of CNPs lies in the fact that it can maintain the characteristics of neural stem cells. The expression of nestin, a specific marker of neural stem cells, was found in CNPs in this study. Nestin is expressed only in the embryonic neuroepithelial cells and disappears after birth, so it is a specific marker of neural stem cells.^25^ There is no correlation between the differentiation and proliferation of hair cells.^26^ Cell proliferation and differentiation belong to two different directions of cell division. CNPs mainly undergo cell proliferation through mitosis, so it can be detected with Brdu (5-bromodeoxyuracil nucleoside). The results of Brdu detection in this study reveal that RA cannot promote the proliferation of CNPs; on the contrary, it inhibits the cell proliferation, which further supports the conclusion that CNPs can differentiate into hair cells.

In conclusion, CNPs can differentiate into hair cells marked by the expressions of Math1 and myosinVIIa under the induction of RA, which has positive significance for the study of hair cell regeneration.

The number of patients with sensorineural hearing loss is increasing all over the world. It is still an important subject for deaf rehabilitation research in China and abroad to explore treatment methods that meet more physiological conditions and requirements. Research on hair cell regeneration or hair cell transplantation is a hot topic in this field; although many studies have been carried out in China and abroad, many basic issues remain to be solved, such as whether the regeneration of hair cells can be accompanied by functional recovery or only by changes in morphology and marker indicators. Specific issues, such as how hair cell transplantation can overcome low survival rate or cannot survive in the recipient, still require further studies.

## Conclusion

The inner ear progenitor cells are undifferentiated during the passaging process, so they can maintain the characteristics of neural stem cells; the proliferation of inner ear progenitor cells is inhibited under RA induction, and certain parts of them differentiate into hair cells. The markers of hair cells (Math1 and myosinVIIa) can be detected at gene and protein levels.

## Conflicts of interest

The authors declare no conflicts of interest.
